# Predictors of Success after Extracorporeal Shock Wave Lithotripsy (ESWL) for Renal Calculi Between 20—30 mm: A Multivariate Analysis Model

**DOI:** 10.1100/tsw.2006.370

**Published:** 2006-03-23

**Authors:** Ahmed El-Assmy, Ahmed R. El-Nahas, Mohamed E. Abo-Elghar, Ibrahim Eraky, Mahmoud R. El-Kenawy, Khaled Z. Sheir

**Affiliations:** ^1^ Urology & Nephrology Center, Urology Department, Mansoura University, Mansoura, Egypt; ^2^ Radiology Department, Mansoura University, Mansoura, Egypt

**Keywords:** shock wave lithotripsy, renal calculi, prognostic factors

## Abstract

The first-line management of renal stones between 20—30 mm remains controversial. The Extracorporeal Shock Wave Lithotripsy (ESWL) stone-free rates for such patient groups vary widely. The purpose of this study was to define factors that have a significant impact on the stone-free rate after ESWL in such controversial groups. Between January 1990 and January 2004, 594 patients with renal stones 20—30 mm in length underwent ESWL monotherapy. Stone surface area was measured for all stones. The results of treatment were evaluated after 3 months of follow-up. The stone-free rate was correlated with stone and patient characteristics using the Chi-square test; factors found to be significant were further analyzed using multivariate analysis.Repeat ESWL was needed in 56.9% of cases. Post-ESWL complications occurred in 5% of cases and post-ESWL secondary procedures were required in 5.9%. At 3-month follow-up, the overall stone-free rate was 77.2%. Using the Chi-square test, stone surface area, location, number, radiological renal picture, and congenital renal anomalies had a significant impact on the stone-free rate. Multivariate analysis excluded radiological renal picture from the logistic regression model while other factors maintained their statistically significant effect on success rate, indicating that they were independent predictors. A regression analysis model was designed to estimate the probability of stone-free status after ESWL. The sensitivity of the model was 97.4%, the specificity 90%, and the overall accuracy 95.6%.Stone surface area, location, number, and congenital renal anomalies are prognostic predictors determining stone clearance after ESWL of renal calculi of 20—30 mm. High probability of stone clearance is obtained with single stone ≤400 mm2 located in renal pelvis with no congenital anomalies. Our regression model can predict the probability of the success of ESWL in such controversial groups and can define patients who would need other treatment modality.

### INTRODUCTION

The first question in the management of any stone is whether the situation is amenable to Extracorporeal Shock Wave Lithotripsy (ESWL); this because of the ease of use and noninvasiveness of the procedure. The real issue is whether the excellent results obtainable with this technology in patients with small stones translate into successful treatment when the targets are large stones.

Stone burden remains the primary factor in deciding the appropriate treatment for a patient with kidney calculi[1]. Calculi smaller than 20 mm are still largely treated with ESWL as the first-line management[2]. Currently, the disadvantages of ESWL for large complex stones are clearly established; the results of ESWL treatment of calculi larger than 30 mm are dismal and should be avoided[3].

The first-line management of renal stones between 20–30 mm remains controversial. Lingeman and associates[4] showed that the frequency of multiple treatments increased from 10 to 33% when ESWL was used to treat stones of 1–2 and 2–3 cm, respectively. In addition, stone-free rate was only 34%, compared with 90% in percutaneous nephrolithotomy (PNL)–treated patients[5]. High retreatment rates and the need for auxiliary procedures were the basis for the NIH Consensus Conference recommendation that patients with stones larger than 2 cm should be approached with PNL initially, followed, if needed, by ESWL[2]. However, because ESWL stone-free rates for renal stones of 2–3 cm range widely (33–65%)[2], ESWL may still be considered an option if the patient is advised about the higher retreatment rate and the lower likelihood of achieving a stone-free state in comparison with PNL[6].

Few studies reported the prognostic factors of ESWL in large renal calculi[7,8,9]. However, predictors of success of ESWL in patients with renal calculi of 20–30 mm that would differentiate between those who should be treated by ESWL and those who should undergo other treatment modalities are yet to be definitively determined. Also, a common problem in the previous studies was that they relied on measuring the longest diameter of the stone, which may be misleading in estimating the stone burden. Moreover, these studies utilized only univariate statistical analysis that does not take into account the possible interactions between the examined variables.

To overcome these limitations, we reviewed our experience with ESWL monotherapy in treating patients with renal calculi between 20–30 mm using stone surface area as a more careful estimate of the stone burden[10]. Also, we used multivariate analysis to design a logistic regression model that can predict the probability of stone-free status and will help to select the ideal patient in such controversial groups for ESWL in a prospective manner with exclusion of any patients who would be best managed by PNL.

### MATERIALS AND METHODS

#### Patients

The computerized patients' records at our institute were reviewed to identify patients who underwent ESWL monotherapy for renal stones 20–30 mm in length, between January 1990 and January 2004. In all, 594 consecutive patients with a mean (SD, range) age of 42.5 years (11, 6–74) were identified. This retrospective study included 431 (72.6%) males and 163 (27.4%) females. Pre-ESWL evaluation included history and clinical examinations, plain abdominal X-ray (KUB) and ultrasonography (US). Excretory urography (IVU) was performed if the serum creatinine was <2 mg/dl. Laboratory investigations included urinalysis, urinary culture and sensitivity tests, serum creatinine, and a coagulation profile. Patients with positive urine cultures were treated with the appropriate antibiotics prior to ESWL. Among our patients, 26 (4.4%) had congenital renal anomalies: 11 horseshoe, 12 malrotated, and 3 duplex kidneys.

### Stones

All the treated stones were 20–30 mm in length. Stone burden was determined by measuring stone surface area on the pre-ESWL X-rays as first reported by Lam et al.[10]. The pre-ESWL images were achieved at Integrated Patient Information System (General Electric Medical System, Milwaukee, WI). Then we used magic view workstation to draw a manual region of interest around the stone after measurement calibration. The surface area of the stone was then calculated automatically in square millimeters. The mean surface area of stones was 346 ± 141 $mm^2$ (range 100–900 $mm^2$). Stone analysis was carried out for 164 patients with X-ray diffraction[11].

### Technique

All patients underwent ESWL using the Dornier MFL 5000 lithotripter (Dornier MedTech GmbH, Germering, Germany). Pre-ESWL ureteric JJ stents were placed in 55 patients (9.3%). Indications for ureteral stenting were solitary kidneys in 15 patients (2.5%), calcular anuria in 5 (0.8%), and significant hydronephrosis in the remaining 35 patients (6.4%). All patients received sedoanalgesia in the form of meperidine hydrochloride (1 mg/kg) and/or fentanyl (1.5 µg/kg). Therapy was usually started at a low power of 14 kV and then it was increased gradually to 24 kV. The interval between sessions was not less than 1 week. A total of 3,000 shocks were delivered for each session or until complete fragmentation of the stone.

### Follow-Up

Patients were reviewed 1 week after each session using KUB and renal US to assess fragmentation and the presence of renal obstruction. Repeat treatment was carried out if inadequate fragmentation of the stone was observed. If there was no breakage of the stone after three sessions, the case was considered an ESWL failure. Patients were finally evaluated 3 months after last lithotripsy session by KUB and US or spiral computerized tomography (CT) in faint opaque stones. Success was defined as complete clearance of the stones with no residual fragments. Noninfected, asymptomatic gravels less than 4 mm were considered insignificant.

### Statistical Analysis

Patient and stone characteristics were correlated to the stone-free rate using the Chi-square test. Factors found to be significant were further analyzed using multivariate analysis (stepwise logistic regression analysis with backward elimination using the likelihood ratio) to identify those that acted independently and to predict the probability of stone-free status after ESWL[12].

## RESULTS

Multiple ESWL treatment sessions were required in 338 patients (56.9%). Complete stone fragmentation was achieved after one session in 256 (43.1%) patients, two sessions in 140 (23.6%), three sessions in 96 (16.2%), and more than three sessions in 60 (10.1%). Failure to break the stones after three sessions was recorded in 42 cases (7%) and was managed by PNL. The number of sessions ranged from one to six per case with a median of two. The mean number of shocks per case delivered in several sessions was 6140 ± 3477 and the kilovoltage ranged from 18–24 (median 22 kV).

Post-ESWL complications are listed in Table 1. Steinstrasse were recorded in 65 cases (11%) and passed spontaneously in 39 (60%). Complicated steinstrasse (26 cases) were treated with ureteroscopy and extraction of the leading stone in 20 cases; in the remaining 6 cases, the fragments passed spontaneously after decompression of the obstructed renal units by means of JJ stents in 4 and PCN in 2. Post-ESWL secondary procedures are listed in Table 2. In the present study, an efficiency quotient (EQ) of 0.46 could be achieved using the following formula: EQ = percentage of stone-free patients/(100% + retreatment percentage + percentage of post-ESWL auxiliary procedures)[13].

**TABLE 1**
**Post-ESWL Complications**

| *Complications* | *No.* | *(%)* |
|---|---|---|
| Subcapsular hematoma | 1 | 0.2 |
| Fever (Temp. > 38.5°C) | 3 | 0.5 |
| Complicated steinstrasse | 26 | 4.4 |
| Total | 30 | 5 |

**TABLE 2**
**Post-ESWL Secondary Procedures**

| *Secondary Procedures* | *No.* | *(%)* |
|---|---|---|
| JJ stent placement | 4 | 0.7 |
| PCN | 2 | 0.3 |
| PNL | 9 | 1.5 |
| URS | 20 | 3.4 |
| Total | 35 | 5.9 |

The follow-up data at 3 months were available for all patients. The presence of residual fragments (RFs) was observed in 93 cases (15.6%). The RFs were classified into two groups: significant residual fragments (SRFs; >4 mm) in 29 cases (4.9%) and clinically insignificant residual fragments (CIRFs; ≤4 mm) in 64 (10.8%). Of the 29 patients with SRFs, 20 (3.4%) needed repeat ESWL, the remaining 9 (1.5%) being treated by PNL.

Stone analysis was done for 164 cases and revealed calcium oxalate monohydrate in 41 (25%), calcium oxalate dihydrate in 14 (8.6%), and mixed calcium stones in 109 (66.4%) (mixed with hydroxyl apatite and/or uric acid and/or struvite in different proportions). The success of ESWL monotherapy in relation to stone composition in our series showed no significant difference ($p = 0.43$).

The stone-free rate was correlated with patient characteristics and stone features using the Chi-square test (Table 3). Of the 11 prognostic factors studied, 5 had a significant impact on success rate, namely the site, number, and surface area of the stones; the radiological renal picture; and congenital renal anomalies. These 5 factors were further analyzed using a logistic regression model, which resulted in the exclusion of the radiological renal picture from the model, while the other prognostic factors maintained their statistically significant effect on ESWL outcome, indicating that they acted independently (Table 4).

Using the regression model, we can define the stone-free ratio of a certain category in comparison to the reference category (Table 4). For example, the probability of stone-free status is 2.8 times greater for patients with stones ≤400 $mm^2$ in surface area in comparison to patients with stones >400 $mm^2$.

**TABLE 3**
**Factors Affecting Stone Clearance**

| | *Stone Free (n = 459)* | | *Residual Stones (n = 135)* | | |
|---|---|---|---|---|---|
| | **No.** | **%** | **No.** | **%** | ***p*-value*** |
| **Age (years)** | | | | | |
| ≤40 | 213 | 79.2 | 56 | 20.8 | 0.32 |
| >40 | 246 | 75.7 | 79 | 24.3 | |
| **Sex** | | | | | |
| Male | 329 | 76.3 | 102 | 23.7 | 0.44 |
| Female | 130 | 79.8 | 33 | 20.2 | |
| **Kidney morphology** | | | | | |
| -Perfect | 241 | 81.4 | 55 | 18.6 | 0.03 |
| -Hydronephrotic | 190 | 74.2 | 66 | 25.8 | |
| -Pyelonephritic | 28 | 66.7 | 14 | 33.3 | |
| **Stone surface area ($mm^2$)** | | | | | |
| ≤ 400 | 362 | 83 | 74 | 17 | <0.001 |
| > 400 | 97 | 61.4 | 61 | 38.6 | |
| **Stone side** | | | | | |
| Right | 228 | 75.7 | 73 | 24.3 | 0.38 |
| Left | 231 | 78.8 | 62 | 21.2 | |
| **Stone site** | | | | | |
| Upper calyx | 37 | 82.2 | 8 | 17.8 | <0.001 |
| Middle calyx | 17 | 70.8 | 7 | 29.2 | |
| Lower calyx | 81 | 69.8 | 35 | 30.2 | |
| Pelvis | 216 | 87.8 | 30 | 12.2 | |
| Multiple sites | 104 | 63.8 | 59 | 36.2 | |
| **Stone nature** | | | | | |
| De-novo | 361 | 77.5 | 105 | 22.5 | 0.81 |
| Recurrent | 98 | 76.6 | 30 | 23.4 | |
| **Opacity** | | | | | |
| Opaque | 442 | 77 | 132 | 23 | 0.58 |
| Faint-opaque | 17 | 85 | 3 | 15 | |
| **Stone number** | | | | | |
| Single | 291 | 85.6 | 49 | 14.4 | <0.001 |
| Multiple | 168 | 66.1 | 86 | 33.9 | |
| **Congenital anomalies** | | | | | |
| No | 446 | 78.5 | 122 | 21.5 | 0.003 |
| Yes | 13 | 50 | 13 | 50 | |
| **Pre-ESWL JJ stenting** | | | | | |
| No | 420 | 77.9 | 119 | 22.1 | 0.24 |
| Yes | 39 | 70.9 | 16 | 19.1 | |

The equation for logistic regression is Z = constant + B for stone(s) surface area + B for stone(s) site + B for stone(s) number + B for congenital renal anomalies where Z is the linear combination of the variables, B is the regression coefficient and –1.242 is the constant of the model. The probability of the patient being stone-free is $1/(1 + e^{-z})$, where e is the base of the natural logarithm (= 2.718). If the estimated probability is >0.5, we predict that the patient will be free of stones. If the probability is <0.5, we predict that the patient will have residual stones. For example, if we have a patient with a single stone ≤400 $mm^2$ in surface area in the renal pelvis with no congenital renal anomalies Z = –1.242 + 0.640 + 1.042 + 0.740 + 1.268 = 2.448. The probability of the patient being stone-free = 0.92. The sensitivity of the model was 97.4%, the specificity 90%, and the overall accuracy 95.6%.

**TABLE 4**
**Prognostic Factors Maintained in Stepwise Logistic Regression**

| *Variables* | *Regression Estimate (B)*[a] | *Standard Error (SE)* | *Relative Risk (95% CI) EXP (B)*[b] *(Lower, Upper)* | p-*value* |
|---|---|---|---|---|
| **Stone surface area (mm$^2$)** | | | | |
| ≤400 | 1.042 | 0.223 | 2.835 (1.831–4.389) | <0.001 |
| >400 (Reference) | 0 | 0 | 1 | |
| **Stone number** | | | | |
| Single | 0.640 | 0.273 | 1.896 (1.109–3.240) | 0.019 |
| Multiple (Reference) | 0 | 0 | 1 | |
| **Stone site** | | | | |
| Upper calyx | 0.326 | 0.455 | 1.386 (0.568–3.383) | 0.474 |
| Middle calyx | –0.174 | 0.531 | 0.840 (0.297–2.379) | 0.743 |
| Lower calyx | –0.106 | 0.311 | 0.900 (0.489–1.653) | 0.733 |
| Pelvis | 0.740 | 0.336 | 2.096 (1.085–4.491) | 0.028 |
| Multiple site (Reference) | 0 | 0 | 1 | |
| **Congenital anomalies** | | | | |
| No | 1.268 | 0.440 | 3.554 (1.499–8.427) | 0.004 |
| Yes (Reference) | 0 | 0 | 1 | |

[a] Regression coefficient.

[b] Stone-free rate when the category of a certain variable is compared to the reference category.

### DISCUSSION

First-line management for renal stones between 20 and 30 mm is somewhat controversial and the ESWL stone-free rates for such groups range widely[2]. This difference could be explained by the fact that these authors relied on measuring the longest diameter of the stone, which may be misleading in estimating the stone burden**.** Measurement of stone surface area is an accurate and reproducible method to quantify stone burden[10]. Lam and associates stratified the ESWL results in treating staghorn calculi based on the surface area[10]. Stone surface area had an inverse correlation with stone-free results. These investigators reported the following surface area/stone-free rate stratification: 0–500 mm$^2$, 63.2%; 501–1000, 45.7%, 1001–1500, 22.2%. In our study, the stone-free rates were 83 and 61.4% for stones ≤400 and >400 mm$^2$, respectively ($p < 0.001$). When the surface area was evaluated in association with other variables in the logistic regression analysis, it remained as one of the strongest factors associated with stone-free status in this group of patients.

For upper and middle calyceal stones, stone-free rates range from 70–90%, whereas those for lower calyceal calculi range between 50 and 70%[14]. In the present study, the lower calyceal and multiple site calculi had the lowest clearance rate (69.8 and 63.8%, respectively). The probability of success of ESWL is doubled for stones located in the renal pelvis in comparison to multiple site stones ($p = 0.028$).

In our study, the probability of stone-free status was 1.9 times greater for patients with single compared to multiple stones ($p = 0.019$). Ackermann et al.[15] reported that stone number was one of the significant predictors influencing treatment outcome after ESWL using multivariate analysis.

Horseshoe kidney is the most common congenital fusion renal anomalies. A common finding is the high insertion of the ureter into an elongated anteriorly rotated renal pelvis resulting in impaired urinary drainage. The results of ESWL for horseshoe kidney stones vary widely and stone-free rates between 28 and 78% have been reported[16,17]. In our series, the probability of stone-free status was 3.5 times greater for patients with normal kidneys compared to those with congenital renal anomalies ($p = 0.004$).

In a single variable analysis, we found that obstructed and pyelonephritic kidneys had a significantly lower stone-free rate compared with normal kidneys ($p = 0.03$). This may be due to weak peristalses that lead to poor clearance of the fragments. In a recent study of 680 patients with lower pole calculi, Poulakis et al.[18] reported that the pattern of dynamic urinary transport represented the most influential predictor of stone clearance.

Multiple authors have reported that ESWL fragility varies between different stone compositions and even within stones of the same composition[19,20]. Cystine and brushite are the most ESWL-resistant stones followed in descending order by calcium oxalate monohydrate, hydroxyapatite, struvite, calcium oxalate dihydrate, and uric acid[20]. However, stone analysis did not significantly affect the ESWL outcome in our series due to the fact that all the stones analyzed in our series were calcium-based stones.

Many investigators believe that pre-ESWL JJ stenting of large renal stones helps to prevent obstruction and facilitates passage of fragments[8,21]. On the other hand Ryan et al.[22] showed that *in situ* ureteric stents impair ureteric peristalsis and/or trap large fragments, thus delaying stone clearance. In our study, we found that pre-ESWL JJ stents did not significantly affect the incidence of neither complications nor stone clearance. However, since the use of stents in our study was not randomized, it is difficult to conclude that stents would not have helped. The patients who got stents had a larger stone burden, the mean stone surface areas were 447 and 336 $mm^2$ for stented and nonstented patients, respectively ($p < 0.001$). Therefore, comparing the use of additional procedures and complications in stented group with those more straightforward patients who did not get stents is inappropriate.

In all studies that investigated the factors affecting ESWL outcome in management of large renal stones[7,8,9,10], a univariate analysis was only utilized, which does not take into account the possible interactions between the examined variables. To overcome this serious limitation, one has to resort to multivariate statistical techniques. The most commonly used method is the logistic regression analysis[12].

To the best of our knowledge, we report the largest series evaluating the outcome of ESWL monotherapy for renal stones of 20–30 mm. Also, this is the first report utilizing the stone surface area and multivariate analysis in evaluation of ESWL outcome in such cohort of patients. Our results of logistic regression model indicate that there are four factors that influence the outcome of ESWL independently and significantly: the surface area, site, number of stones, and congenital renal anomalies. Our model had an accuracy of 95.6% for the prediction of stone-free status. This model helps to choose patients with such calculi suitable for ESWL in a prospective manner and identify patients who would be best treated by endoscopic techniques.

Finally, in the era of CT, some authors prefer to use it for estimation of stone volume and avoid the use of IVU[23]. We preferred to use the surface area measured form the pre-ESWL KUB and IVU films, because in the developing world, we have a large volume of stone patients from very poor socioeconomic background. The economics of health care is a major concern, especially when many patients have to self-finance their treatment with limited resources. KUB, US, and IVU are modalities on which most urologists base their diagnosis and therapeutic decisions, and will continue to do so for some time.

### CONCLUSIONS

The surface area, site, number of stones, and congenital renal anomalies are the most important prognostic factors affecting stone clearance of renal calculi of 20–30 mm after ESWL monotherapy. Best ESWL results are obtained with single stone $\leq 400$ $mm^2$ located in the renal pelvis with absence of congenital renal anomalies. Our regression analysis model can predict the probability of stone-free status with an accuracy of 96% and helps in prospective selection of patients who will respond well to ESWL and exclusion of any patient who would be best treated using endoscopic maneuvers.
